# Silviridoside: A New Triterpene Glycoside from *Silene viridiflora* with Promising Antioxidant and Enzyme Inhibitory Potential

**DOI:** 10.3390/molecules27248781

**Published:** 2022-12-11

**Authors:** Markhabo M. Makhmudova, Markus Bacher, Gokhan Zengin, Thomas Rosenau, Fadia S. Youssef, Diena M. Almasri, Sameh S. Elhady, Nilufar Z. Mamadalieva

**Affiliations:** 1 Institute of the Chemistry of Plant Substances of the Academy Sciences of Uzbekistan, Tashkent 100170, Uzbekistan; 2 Department of Chemistry, Institute of Chemistry of Renewable Resources, University of Natural Resources and Life Sciences Vienna (BOKU), 3430 Tulln, Austria; 3 Department of Biology, Science Faculty, Selcuk University, Konya 42130, Turkey; 4 Department of Pharmacognosy, Faculty of Pharmacy, Ain-Shams University, Abbasia, Cairo 11566, Egypt; 5 Department of Pharmacy Practice, Faculty of Pharmacy, King Abdulaziz University, Jeddah 21589, Saudi Arabia; 6 Department of Natural Products, Faculty of Pharmacy, King Abdulaziz University, Jeddah 21589, Saudi Arabia

**Keywords:** *Silene viridiflora*, triterpene glycoside, silviridoside, antioxidant, enzyme inhibitor, ADME/TOPKAT, drug discovery, sustainability of natural resources

## Abstract

A new triterpene glycoside, silviridoside, was isolated from the aerial parts of *Silene viridiflora* (Caryophyllaceae) using different chromatographic techniques. The structure of silviridoside was comprehensively elucidated as 3-*O-*β-D-galacturonopyranosyl-quillaic acid 28-*O*-β-D-glucopyranosyl-(1→2)-[α-L-rhamnopyranosyl-(1→3)]-β-D-fucopyranosyl ester by one- and two-dimensional nuclear magnetic resonance (NMR) spectroscopy and high-resolution mass spectrometry (HR-MS). Silviridoside showed promising antioxidant activity in different antioxidant assays such as 2,2-diphenyl-1-picrylhydrazyl (DPPH) (2.32 mg TE/g), 2,2′-azino-bis(3-ethylbenzothiazoline-6-sulfonic acid) (ABTS) (1.24 mg TE/g), cupric-reducing antioxidant capacity (CUPRAC) (9.59 mg TE/g), ferric-reducing antioxidant power (FRAP) (5.13 mg TE/g), phosphomolybdenum (PHD) (0.28 mmol TE/g), and metal-chelating (MCA) (6.62 mg EDTA/g) assays. It exhibited a good inhibitory potential on acetylcholinesterase (AChE) (2.52 mg GALAE/g), butyrylcholinesterase (BChE) (7.16 mg GALAE/g), α-amylase (0.19 mmol ACAE/g), α-glucosidase (1.21 mmol ACAE/g), and tyrosinase (38.83 mg KAE/g). An in silico evaluation of the pharmacodynamic, pharmacokinetic, and toxicity properties of silviridoside showed that the new compound exhibited reasonable pharmacodynamic and pharmacokinetic properties without any mutagenic effect, but slight toxicity. Thus, it could be concluded that silviridoside could act as a promising lead drug for pharmaceutical and nutraceutical developments to combat oxidative stress and various disorders, but a future optimization is necessary.

## 1. Introduction

Free radicals, naturally produced within the human body, trigger many adverse effects and cause oxidative damage in proteins, lipids, and genetic material, which is counteracted by the human antioxidant system. An uncontrolled balance between the antioxidant defense and the production of free radicals results in undesirable side effects [1]. Antioxidants from natural products can restore this balance, reducing oxidative stress and its undesired health effects such as nervous disorders and hyperglycemia [2,3,4,5]. Nowadays, enzyme inhibitors are recognized as targets to treat a variety of diseases, including cancer, diabetes, hypertension, cardiac disorders, and Alzheimer’s [6]. Cholinesterase inhibitors are widely used to treat neurodegenerative disorders such as Alzheimer’s disease, which is characterized by an irreversible neurological pattern [7,8]. In addition, tyrosinase is crucial for the formation of melanin, which leads to the overproduction of skin pigments and the appearance of dark spots in different skin areas [9,10]. The inhibition of α-amylase and α-glucosidase is widely used to reduce postprandial glucose levels and, consequently, to control postprandial hyperglycemia in diabetic patients [11,12,13]. The discovery of novel enzyme inhibitors could open a new window for treating diseases.

Natural product-based drugs are highly recommended all over the globe due to their safer properties with a reasonably high activity compared with synthetic drugs [14]. Natural products provide a wide array of secondary metabolites to which their promising biological activity is also attributed [15]. *Silene* is a genus of flowering plants in the Caryophyllaceae family with about 700 species that are spread in Eurasia, America, and Africa [16]. *Silene viridiflora* L. natively grows in Turkey; Central, West, and South Europe; East Asia; and Crimea–Siberia [17]. Previous phytochemical studies of *S. viridiflora* resulted in the isolation and identification of several ecdysteroids [18], lipids [19], essential oils [20], carbohydrates, and microelements [21]. Among the isolated ecdysteroids from *S. viridiflora*, the principal active component is 20-hydroxyecdysone, which has shown immunomodulatory, cytoprotective, and adaptogenic potential [22,23].

Continuing the search for structurally unique and biologically active compounds, the aerial parts of *S. viridiflora* were investigated in this work. As a result, one new triterpene glycoside-denominated silviridoside was isolated using different chromatographic techniques, comprehensively elucidated using advanced spectroscopic techniques such as one- and two-dimensional nuclear magnetic resonance (1D and 2D NMR) and high-resolution mass spectrometry (HR-MS) and tested in different antioxidant and enzyme inhibition assays. In addition, an evaluation of its ADMET characteristics and a TOPKAT (Toxicity Prediction using Komputer-Assisted Technology) prediction were performed in silico using Discovery Studio 4.5 software (Accelrys Inc., San Diego, CA, USA) to predict its pharmacodynamic, pharmacokinetic, and toxicity properties.

## 2. Results and Discussion

### 2.1. Isolation and Structural Elucidation of Compound **1**

A phytochemical investigation performed on the methanol extract of the aerial parts of *S. viridiflora* resulted in the isolation of compound **1**, which was further subjected to a comprehensive structural elucidation using several chromatographic and spectroscopic methods. The molecular formula of compound **1** was established as C_54_H_84_O_24_, based on the HR-ESI-MS analysis, with a molecular ion peak at *m*/*z* 1115.5304 [M-H]^−^ (calculated for C_54_H_83_O_24_: 1115.5274). The ^1^H NMR spectrum (data in Table 1) showed the presence of six tertiary methyl groups at δ_H_ 0.65, 0.84, 0.91 (× 2), 0.98, and 1.32 ppm. A combined analysis of the ^1^H, ^13^C, and HSQC spectra also revealed a trisubstituted double bond with the olefinic proton at δ_H_ 5.22 ppm and its corresponding carbon at δ_C_ 121.2 ppm as well as a quaternary carbon at δ_C_ 143.2 ppm. In addition, the HSQC spectrum also indicated two oxygenated methine groups at δ_H_/δ_C_ 3.82/80.2 ppm and δ_H_/δ_C_ 4.34/72.4 ppm. This information pointed to compound **1** featuring an olean-12-ene skeleton [24]. Another striking feature was the presence of an aldehyde function at δ_H_/δ_C_ 9.35/207.0 ppm. This aldehyde function was assigned to position 23 based upon the HMBC correlations of methyl protons (CH_3_-24) at δ_H_ 0.98 ppm and the quaternary C-5 carbon at δ_C_ 46.7 ppm. Moreover, a long-range cross-peak from CH_3_-24 to the carbon (C-3) at δ_C_ 80.2 ppm established oxygenation at position 3. The second oxymethine function (δ_H_/δ_C_ 4.34/72.4) was assigned to position 16 due to COSY correlations with H-15 (δ_H_ 1.64 and 1.26 ppm), which further showed long-range cross-peaks to C-27 (δ_C_ 26.4 ppm). The stereochemistry at the C-3 and C-16 chiral centers was determined via NOESY correlations; a cross-peak from H-3 (δ_H_ 3.82 ppm) to H-5 (δ_H_ 1.25 ppm) led to H-3α whereas a correlation between H-16 (δ_H_ 4.34 ppm) and CH_3_-26 (δ_H_ 0.65 ppm) established a β-orientation of H-16. Based on the above evidence, the skeleton (aglycon part) of compound **1** was established to be the triterpene quillaic acid. This was further confirmed by the COSY, NOESY, HSQC, and HMBC spectra, along with a comparison with the literature data [24].

The ^1^H NMR spectrum of compound **1** showed four resonances (Table 2) from the anomeric protons at δ_H_ 4.24, (d, *J* = 7.7 Hz), 5.42 (*br, s*), 3.98 (d, *J* = 7.6 Hz), and 5.22 (d, *J* = 8.0 Hz), which showed HSQC correlations with four anomeric carbon atoms at δ_C_ 105.90, 99.39, 102.90, and 93.03, respectively. The complete assignments of the carbohydrate signals were accomplished by an extensive analysis of the different 2D NMR spectra (COSY, TOCSY, HSQC, HMBC, and HSQC–TOCSY). Based on this analysis, one β-D-galacturonic acid unit (GalA), one *α*-rhamnopyranosyl (Rha), one β-D-glucopyranosyl (Glc), and one β-D-fucopyranose (Fuc) were identified. Moreover, the NOESY cross-peak between δ_H_ 3.98 (d, *J* = 7.6 Hz, H-1′ of GalA) and δ_H_ 3.82 (m, H-3), along with the HMBC correlations of 3.98 (H-1′) with C-3, established the β-D -galacturonic acid unit at C-3 of the aglycone. Furthermore, the HMBC correlation between δ_H_ 5.22 (d, *J* = 8.0 Hz, Fuc, H-1′′′′) and δ_H_ 175.0 (C-28) confirmed the attachment of the *β*-D-fucopyranose unit to C-28. HMBC correlations between δ_H_ 3.53 (*m*, Fuc-3, H-3′′′′) and δ_C_ 99.39 (Rha-1, H-1′′), and between δ_H_ 5.42 (*br, s,* Rha-1, H-1′′) and δ_C_ 74.38 (Fuc-3, C-3′′′′) proved the (1→3) linkage between the fucose and rhamnose units. This was further supported by the NOESY correlations between δ_H_ 3.53 (*m*, Fuc-3, H-3′′′′) and δ_H_ 5.42 (*br, s,* Rha-1, H-1′′). Moreover, the HMBC correlations between δ_H_ 3.82 (*m*, Rha-2, H-3′′) and δ_C_ 105.90 (Glc-1, C-1′′′), along with the NOESY correlations between δ_H_ 4.24 (*d*, *J* = 7.7 Hz, Glc-1, H-1′′′) and δ_H_ 3.82 (*m*, Rha-2, H-2′′), confirmed the (1→2) linkage between Glc and Rha. Based upon the above spectroscopic results, the structure of compound **1** was established as 3-O-*β*-D-galacturonopyranosyl-quillaic acid 28-O-β-D-glucopyranosyl-(1→2)-[α-L-rhamnopyranosyl-(1→3)]-β-D-fucopyranosyl ester, which was denominated as silviridoside. The complete ^1^H and ^13^C NMR chemical shifts and coupling constants are listed in Table 1 and Table 2.

**Figure 1:**
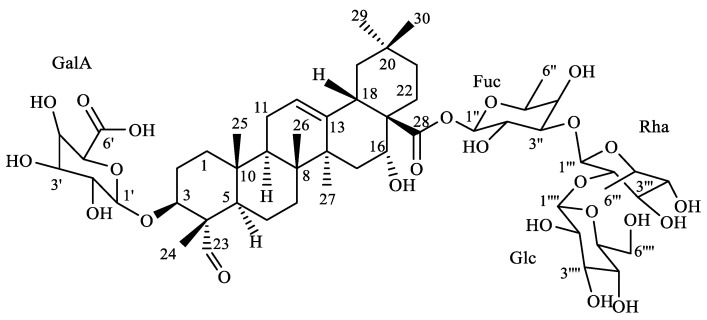
Structure of silviridoside (compound **1**).

Thus, compound **1** represented a new triterpene glycoside, named silviridoside (Figure 1).

**Figure 2:**
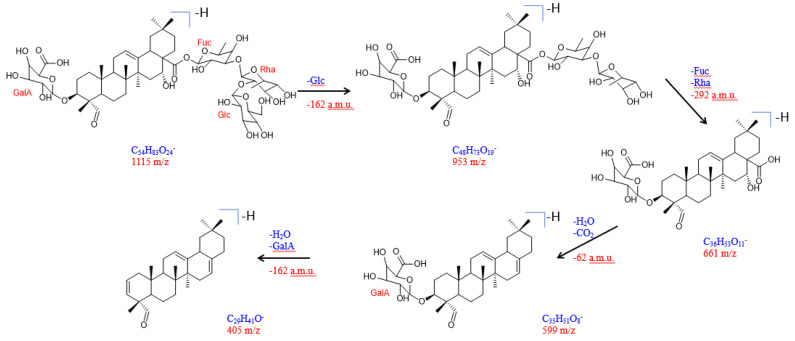
Proposed fragmentation pathway for silviridoside (compound **1**) in ESI-MS/MS negative mode.

The structure and connection of the sugar moieties were also confirmed via the observed fragmentation pattern of the molecule in a negative ESI-MS/MS mode (Figure S2). The proposed fragmentation pathway of compound **1** is presented in Figure 2. A loss of glucose gave the fragment a peak at *m*/*z* 953 whereas, due to the fragmentation of the trisaccharide unit, a peak at *m*/*z* 661 was formed. Further decarboxylation led to a fragment with a peak at *m*/*z* 599. Finally, splitting off the galacturonic acid moiety built a fragment with a peak at *m*/*z* 405.

### 2.2. Biological Evaluation of Compound **1**

The antioxidant and enzyme inhibitory properties of silviridoside were investigated by different spectrophotometric assays. The results are summarized in Table 3, showing that silviridoside exhibited moderate antioxidant properties. The compound was more active in the DPPH radical scavenging assay, showing an antioxidant activity of 2.32 mg TE/g compared with the ABTS radical scavenging assay (1.24 mg TE/g). The compound also had a greater cupric-reducing power (estimated at 9.59 mg TE/g) compared with the ferric-reducing power (5.13 mg TE/g). The results for the phosphomolybdenum and metal-chelating assays were 0.28 mmol TE/g and 6.62 mg EDTA/g, respectively. In accordance with our results, other authors have reported significant antioxidant effects of triterpene glycosides [25,26,27].

Enzyme inhibition represents one of the most popular subjects in the pharmaceutical industry [28]. In this study, the enzyme inhibition properties of silviridoside were tested against cholinesterases (AChE and BChE), tyrosinase, amylase, and glucosidase. The compound exhibited inhibitory effects against all the tested enzymes. The anticholinesterase ability (AChE and BChE) was 2.52 and 7.16 mg GALAE/g, respectively. The compound also showed a good tyrosinase inhibitory property (38.83 mg KAE/g). The α-amylase and α-glucosidase inhibitory potentials, which reflected the possible antidiabetic properties of this compound, were 0.19 and 1.21 mmol ACAE/g, respectively. Significant inhibitory abilities of several triterpene glycosides have been reported in the literature [29,30]; thus, we hope that our results are useful for novel applications in pharmaceutical areas.

### 2.3. In Silico Evaluation of the Pharmacodynamic, Pharmacokinetic, and Toxicity Properties of Silviridoside

**Figure 3:**
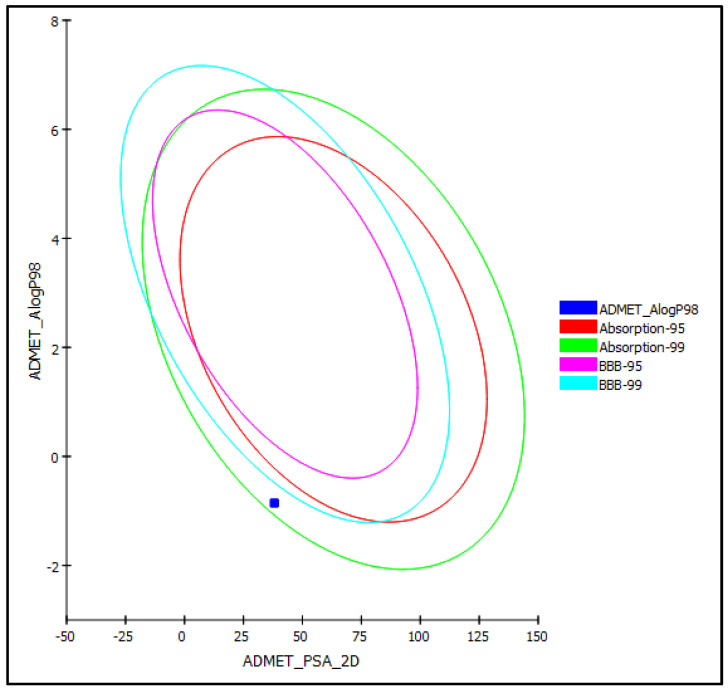
ADMET plot for silviridoside illustrating 95% and 99% confidence limit ellipses corresponding with the blood–brain barrier (BBB) and human intestinal absorption; %; PSA-2D: 2D polar surface area; Alog p98: atom-based log P98.

An in silico evaluation of the pharmacodynamics and pharmacokinetics of silviridoside was performed using the ADMET (absorption, distribution, metabolism, excretion, and toxicity) protocol. Silviridoside was very soluble and showed moderate human intestinal absorption, which placed it within the 99% confidence limit absorption ellipses, as illustrated in Figure 3. In contrast, it showed an unpredictable blood–brain barrier (BBB) penetration and was thus allocated outside the 99% confidence limit BBB ellipses (Figure 3). Silviridoside also revealed less than 90% plasma protein binding (PPB) and a slight toxicity to the liver, but no cytochrome P450 2D6 inhibition. Importantly, the new compound showed optimum cell permeability (polar surface area (PSA) < 140 A° and atom-based log P98 (Alog P98) < 5). The Toxicity Prediction using Komputer-Assisted Technology (TOPKAT) prediction revealed that silviridoside caused no mutagenicity in the Ames mutagenicity test and was non-carcinogenic to female National Toxicology Program (NTP) rats; unfortunately, it caused certain carcinogenic effects in male NTP rats. It also showed a rat oral LD50 and a chronic rat lowest observed adverse effect level (LOAEL) of 1.116 and 0.075 g/kg.bw, respectively. Furthermore, silviridoside showed a mild dermal irritation and a severe ocular irritant effect; thus, it should be handled and used cautiously. The in silico evaluation of the pharmacodynamic, pharmacokinetic, and toxicity properties of silviridoside are summarized in Table 4.

## 3. Materials and Methods

### 3.1. General Experimental Procedures

The NMR experiments were performed using a Bruker Avance II 400 spectrometer (resonance frequencies of 400.13 MHz for ^1^H and 100.63 MHz for ^13^C) equipped with a 5 mm broadband observe probe head with z-gradients at room temperature and standard Bruker pulse programs. The chemical shifts were presented in parts per million (*δ*/ppm) and referenced to residual solvent signals (DMSO-d_6_: 2.49 ppm for ^1^H and 39.6 ppm for ^13^C). The coupling constants (*J*) were reported in Hz. The HR-ESI-MS spectra were recorded on an Orbitrap XEVO G2 Xs QToF mass spectrometer (Waters Inc.) coupled to a UPC^2^ HPLC system (Waters Inc.). Chromatographic separation was performed on a 2-PIC column at 45 °C (2-Picolylamine, Taurus series, Waters Inc., Milford, MA, USA)) using a gradient of supercritical CO_2_ and MeOH with 25 mM ammonium hydroxide. For the data analysis, MassLynx software V4.2 was used. Silica gel (100/200 mesh, Tianjin Sinomed Pharmaceutical, Tianjin, China) and Sephadex LH-20 (GE Healthcare Bio-Sciences AB, Sweden) were used as the stationary media for the column chromatography. Thin layer chromatography (TLC) was performed on aluminum plates pre-coated with silica gel 60 F254 (Merck, Germany).

### 3.2. Plant Materials

The aerial parts (flowers, leaves, and stems) of *S. viridiflora* were collected from the botanical field of the Institute of the Chemistry of Plant Substances (Tashkent, Uzbekistan). The taxonomic authentication was accomplished by Dr. A. Nigmatullaev at the Department of Herbal Plants of the ICPS. The voucher specimen of the plant was deposited in the departmental herbarium under the code 2017/087. The plant material was air-dried and powdered before use.

### 3.3. Extraction and Isolation

The air-dried aerial organs (4 kg) of *S. viridiflora* were ground and then extracted with CH_3_OH (20 L × 3). The extract was condensed to 1 L, diluted with an equal amount of H_2_O, and left overnight. The resulting precipitate was filtered off. The CH_3_OH was evaporated. The aqueous solution was extracted with CHCl_3_ (3 L) and butanol (1 L). The solvents were evaporated in a vacuum to obtain a butanol (168 g) fraction. The dried butanol fraction (80 g) was subjected to silica gel column chromatography (CC) (column size: 11 cm × 90 cm) and the fraction was eluted by CHCl_3_/MeOH gradients with the polarity increasing to 20% MeOH. Each fraction (200 mL) was analyzed by TLC and the fractions with similar TLC patterns were combined to obtain the main fractions (Fr. A–C). Fraction C (3.9 g) was chromatographed over a silica gel column and eluted stepwise by CHCl_3_/MeOH (9:1, *v*/*v* and 4:1, *v*/*v*) and CHCl_3_:MeOH:H_2_O (65:25:4, *v*/*v*/*v*). Ecdysteroid-containing subfractions C9–13 (280 mg) were chromatographed on silica gel (l = 70 cm; d = 3.5 cm) using a system of CHCl_3_:MeOH:H_2_O (70:23:2, *v*/*v*), providing 8 subfractions C9–13/1–8. Subfractions C9–13/5–6 (38 mg) were further separated by Sephadex LH-20 in a mixture of MeOH:water (80:20, *v*/*v*) as the solvent system to obtain compound **1** (9 mg).

### 3.4. Compound Characterization

***Silviridoside* (1):** white amorphous powder, C_54_H_84_O_24_; HR-ESI-MS: *m*/*z*: 1115.5304 [M-H]^−^ (calculated for C_54_H_83_O_24_: 1115.5274). ^1^H NMR (400 MHz, DMSO-d_6_, δ/ppm, and *J*/Hz) and ^13^C NMR (100 MHz, DMSO-d_6_, and δ/ppm) are displayed as Table 1 and Table 2 and Supplementary Materials Figures S1–S11.

### 3.5. Antioxidant Assays

The antioxidant activity was evaluated by free radical scavenging with 2,2-diphenyl-1-picrylhydrazyl (DPPH) as well as 2,2′-azino-bis(3-ethylbenzothiazoline-6-sulfonic acid (ABTS), reducing-power cupric-reducing antioxidant capacity (CUPRAC), ferric-reducing antioxidant power (FRAP), phosphomolybdenum (PHD), and metal-chelating (MCA) assays. The results of these assays were expressed as Trolox equivalents (TE/g extract or TE/g compound). The metal-chelating activity was evaluated as the EDTA equivalent (mg EDTA/g extract or mg EDTA/g compound). The experimental procedures were as previously described [20,31].

### 3.6. Enzyme Inhibition Assays

The enzyme inhibition effects were investigated against acetylcholinesterase (AChE), butyrylcholinesterase (BChE), tyrosinase, α-amylase, and α-glucosidase, as previously described [14]. The inhibitory effects were expressed as standard compound equivalents, with galanthamine used for AChE and BChE, kojic acid for tyrosinase, and acarbose for α-amylase and α-glucosidase.

### 3.7. In Silico Evaluation of Silviridoside

An in silico evaluation of the pharmacodynamics and pharmacokinetics of silviridoside was undertaken according to the ADMET protocol (absorption, distribution, metabolism, excretion, and toxicity) using Biovia Discovery Studio software (Accelrys Inc., San Diego, CA, USA). The ADMET parameters included human intestinal absorption, plasma protein binding (PPB) prediction, blood–brain barrier (BBB) penetration, aqueous solubility, hepatotoxicity level, and inhibition of cytochrome P450 (2D6). The toxicity properties were determined using the TOPKAT protocol where the Ames mutagenicity, rat chronic LOAEL (lowest observed adverse effect level), and rat oral LD_50_, together with ocular and skin irritant effects and carcinogenicity on male and female NTP (National Toxicology Program) rats, were selected as the toxicity descriptors [32,33].

### 3.8. Statistical Analysis

The results of each assay were reported as the means ± SD (n = 3) of three parallel measurements. The calculation for each in vitro assay was performed using GraphPad version 9.2.

## 4. Conclusions

In the current study, a new triterpene glycoside—silviridoside—was isolated from the aerial parts of *Silene viridiflora*. The structure of the new compound was comprehensively identified using different spectroscopic techniques, comprising HR-ESI-MS and 1D and 2D NMR spectroscopy. Different antioxidant assays such as 2,2-diphenyl-1-picrylhydrazyl (DPPH) and 2,2′-azino-bis(3-ethylbenzothiazoline-6-sulfonic acid (ABTS), cupric-reducing antioxidant capacity (CUPRAC), ferric-reducing antioxidant power (FRAP), phosphomolybdenum (PHD), and metal-chelating (MCA) assays were performed, and confirmed a pronounced activity as an antioxidant. In addition, silviridoside revealed a potent inhibition of enzymes such as acetylcholinesterase (AChE), butyrylcholinesterase (BChE), α-amylase, and tyrosinase. Moreover, an in silico evaluation of the pharmacodynamic, pharmacokinetic, and toxicity properties of silviridoside showed that the new compound exhibited reasonable pharmacodynamic and pharmacokinetic properties without a mutagenic effect, but with a slight toxicity. Thus, it could be concluded that silviridoside is a promising candidate for pharmaceutical and nutraceutical drug developments.

## Figures and Tables

**Table 1:** ^1^H and ^13^C NMR chemical shifts of the aglycone part of compound **1** (DMSO-d_6_, δ/ppm, *J*/Hz, and 400 MHz) and selected NOESY cross-peaks.

No.	δ_C_	δ_H_ (*J*/Hz)	Selected NOESY Cross-Peaks	No.	δ_C_	δ_H_ (*J*/Hz)	Selected NOESY Cross-Peaks
1	37.8	1.01, *m*; 1.58, *m*		16	72.4	4.34, *br,s*	26
2	24.2	1.62, *m*; 1.96, *m*		17	47.9		11, 12
3	80.2	3.82, *m*	5, 1′	18	40.5	2.86, *m*	15a, 19a, 22a, 30
4	54.5			19	46.5	0.99, *m*; 2.25, *m*	
5	46.7	1.25, *m*	3	20	30.2		
6	19.8	0.77, *m*; 1.34, *m*		21	35.0	1.08, *m*; 1.92, *m*	
7	31.8	1.26, *m*; 1.37, *m*		22	30.7	1.63, *m*; 1.79, *m*	
8	41.1			23	207.0	9.35, *s*	2b, 3, 5, 6b, 24, 1′
9	46.1	1.62, *m*		24	9.8	0.98, *s*	
10	35.5			25	15.5	0.91, s	
11	22.9	1.82, *m*		26	16.7	0.65, *s*	12, 16
12	121.2	5.22, *m*		27	26.4	1.32, *s*	
13	143.2			28	175.0		
14	39.4			29	32.9	0.84, *s*	
15	34.8	1.64, *m*; 1.26, *m*		30	24.3	0.91, *s*	

**Table 2:** ^1^H and ^13^C NMR chemical shifts with selected HMBC and NOESY cross-peaks of the sugar moieties of compound **1** (DMSO-d_6_, δ/ppm, *J*/Hz, and 400 MHz).

No.	δ_C_	δ_H_ (*J*/Hz)	Selected HMBC Cross-Peaks	Selected NOESY Cross-Peaks
**GalA**				
**1′**	102.90	3.98, *d* (7.6)	3	3
**2′**	73.71	2.82, *m*		
**3′**	72.16	3.02, *m*		
**4′**	76.89	3.03, *m*		
**5′**	73.81	3.11, *m*	6´	
**6′**	172.61			
**Rhamnose**				
**1″**	99.39	5.42, *br,s*	2″, 3″, 5″, 2″″	3″″
**2″**	81.50	3.82, *m*		1′′′
**3″**	70.23	3.41, *m*		
**4″**	72.47	3.12, *m*		
**5″**	68.59	3.48, *m*		
**6″**	18.16	1.10, *d* (6.1)	4″, 5″	
**Glucose**				
**1′′′**	105.90	4.24, *d* (7.7)	2″	2″
**2′′′**	74.04	3.01, *m*		
**3′′′**	76.50	3.12, *m*		
**4′′′**	70.17	3.03, *m*		
**5′′′**	76.85	3.09, *m*		
**6′′′**	61.32	3.70, *dd* (10.5, 5.8)		
		3.43, *m*		
**Fucose**				
**1″″**	93.03	5.22 *d* (8.0)	28	
**2″″**	74.66	3.55, *m*	4″″	
**3″″**	74.38	3.53, *m*	1″″, 2″″	1″
**4″″**	71.32	3.38, *m*		
**5″″**	70.69	3.58, *m*	1″″	
**6″″**	16.30	1.05, *d* (6.3)	4″″, 5″″	
**OH Groups**				
3″-OH		4.39, *d* (9.3)		
2′-OH		4.46, *d* (4.8)		
6″″-OH		4.61, *t* (5.6)		
4″-OH		4.76, *d* (4.8)		
4’’’-OH		4.75, *m*		
16-OH		4.81, *d* (4.8)		
4″″-OH		4.93, *d* (5.8)		
3″″-OH		4.96, *d* (5.0)		
2’’’’-OH		5.20, *m*		
3′and 4′-OH		4.75		

**Table 3:** Antioxidant and enzyme inhibitory effects of silviridoside.

Antioxidant Activity ^a^	Silviridoside	Enzyme Inhibitory Activity ^b^	Silviridoside
DPPH (mg TE/g)	2.32 ± 0.48	AChE (mg GALAE/g)	2.52 ± 0.48
ABTS (mg TE/g)	1.24 ± 0.29	BChE (mg GALAE/g)	7.16 ± 0.04
FRAP (mg TE/g)	5.13 ± 0.31	Tyrosinase (mg KAE/g)	38.83 ± 0.45
CUPRAC (mg TE/g)	9.59 ± 0.52	Amylase (mmol ACAE/g)	0.19 ± 0.01
PHD (mmol TE/g)	0.28 ± 0.01	Glucosidase (mmol ACAE/g)	1.21 ± 0.01
MCA (mg EDTAE/g)	6.62 ± 0.35		

^a^: Values expressed are means ± SD of three parallel measurements.; TFC: total flavonoid content; PHD: phosphomolybdenum; MCA: metal-chelating; TE: Trolox equivalent; EDTAE: EDTA equivalent. ^b^: Values expressed are means ± SD of three parallel measurements. GALAE: Galatamine equivalent; KAE: kojic acid equivalent; ACAE: acarbose equivalent.

**Table 4:** Absorption, distribution, metabolism, excretion, and toxicity (ADMET/TOPKAT) properties of silviridoside.

Compounds	Silviridoside
**ADMET Descriptors**	
Absorption Level ^a^	1
Solubility Level ^b^	5
BBB Level ^c^	4
PPB Level ^d^	False
CPY2D6	NI
Hepatotoxicity	Toxic
PSA-2D ^e^	38.12
Alog p98 ^f^	−0.86
**TOPKAT Descriptors**	
Ames Prediction	Non-mutagenic
Rat Oral LD50 (g/kg.bw)	1.116
Rat Chronic LOAEL (g/kg.bw)	0.075
Rat Female NTP	Non-carcinogenic
Rat Male NTP	Carcinogenic
Skin Irritancy	Mild
Eye Irritancy	Severe

^a^ 0, 1, 2, and 3 indicate good, moderate, low, and very low absorption, respectively; ^b^ 0, 1, 2, 3, 4, and 5 indicate extremely low, very low but possible, low, good, optimal, and too soluble, respectively; ^c^ 0, 1, 2, 3, and 4 denote very high, high, medium, low, and undefined penetration via BBB, respectively; ^d^ PBB: plasma protein binding and false means less than 90%; ^e^ PSA-2D: 2D polar surface area; ^f^ Alog p98: atom-based log P98; NI: non-inhibitor.

## Data Availability

Data are available in the manuscript.

## Supplementary Materials

The following supporting information can be downloaded at: https://www.mdpi.com/article/10.3390/molecules27248781/s1, Figures S1–S12: NMR and HR-ESI-MS data of silviridoside.
