# Supplementary material for: Silviridoside: A New Triterpene Glycoside from Silene viridiflora with Promising Antioxidant and Enzyme Inhibitory Potential
Source: Molecules. 2022 Dec 11;27(24):8781. doi: 10.3390/molecules27248781 (PMC9785594; doi:10.3390/molecules27248781)
Supplement: Supplementary file 1 [file molecules-27-08781-s001.zip › molecules-2045708-supplementary.pdf]

Silviridoside: A New Triterpene Glycoside from *Silene viridiflora* with Promising Antioxidant and Enzyme Inhibitory Potential

Markhabo M. Makhmudova[1], Markus Bacher[2], Gokhan Zengin[3], Thomas Rosenau[2], Fadia S. Youssef[4], Diena M. Almasri[5], Sameh S. Elhady[6], Nilufar Z. Mamadalieva[1*]

[1] Institute of the Chemistry of Plant Substances of the Academy Sciences of Uzbekistan, Tashkent 100170, Uzbekistan
[2] Department of Chemistry, Institute of Chemistry of Renewable Resources, University of Natural Resources and Life Sciences Vienna (BOKU), 3430 Tulln, Austria
[3] Department of Biology, Science Faculty, Selcuk University, Konya 42130,
[4] Department of Pharmacognosy, Faculty of Pharmacy, Ain-Shams University, Abbasia, Cairo11566, Egypt
[5] Department of Pharmacy Practice, Faculty of Pharmacy, King Abdulaziz University, Jeddah 21589, Saudi Arabia
[6] Department of Natural Products, Faculty of Pharmacy, King Abdulaziz University, Jeddah 21589, Saudi Arabia
* Correspondence: nmamadalieva@yahoo.com

Supplementary data

Figure S1: Representative UHPLC-QToF-MS total ion chromatogram of the methanolic extract isolated from *Silene viridiflora* in negative ionization mode

Figure S2: UHPLC-QToF-MS spectrum of silviridoside in the negative ion mode

Figure S3: UHPLC-QToF-MS/MS spectrum of silviridoside in the negative ion mode

Figure S4: ^{1}H NMR spectrum of silviridoside

Figure S5: ^{1}H NMR spectrum of silviridoside – (Expanded part)

Figure S6: ^{1}H NMR spectrum of silviridoside - (Expanded part)

Figure S7: ^{13}C NMR spectrum of silviridoside

Figure S8: ^{13}C NMR spectrum of silviridoside - (Expanded part)

Figure S9: COSY spectrum of silviridoside

Figure S10: HSQC spectrum of silviridoside

Figure S11: HMBC spectrum of silviridoside

Figure S12: NOESY spectrum of silviridoside

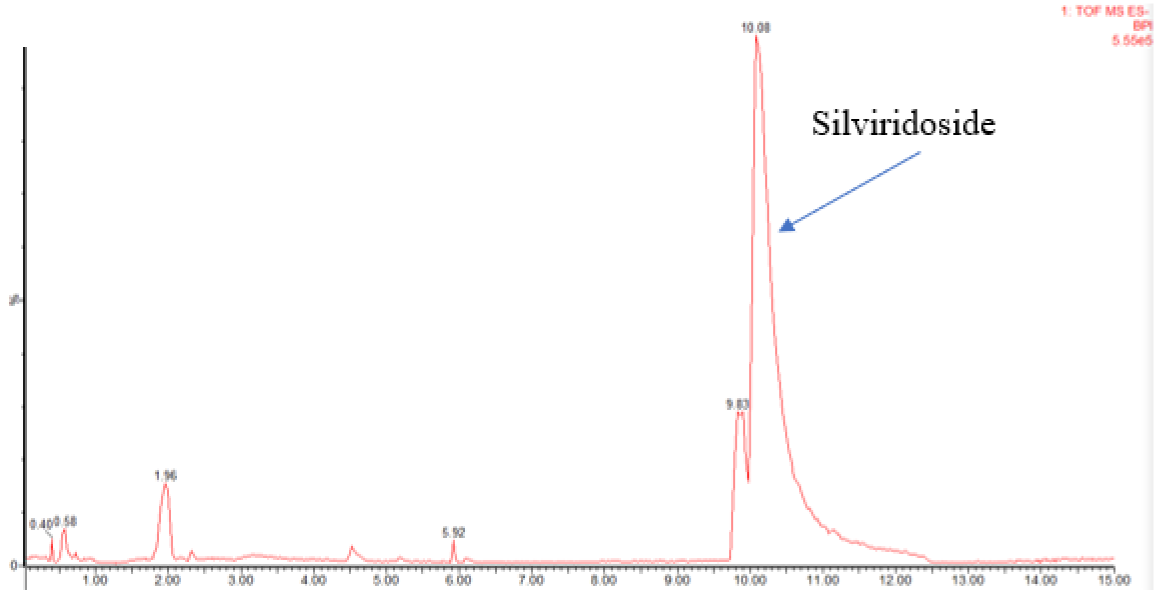

Figure S1: Representative UHPLC-QToF-MS total ion chromatogram of the methanolic extract isolated from *Silene viridiflora* in negative ionization mode

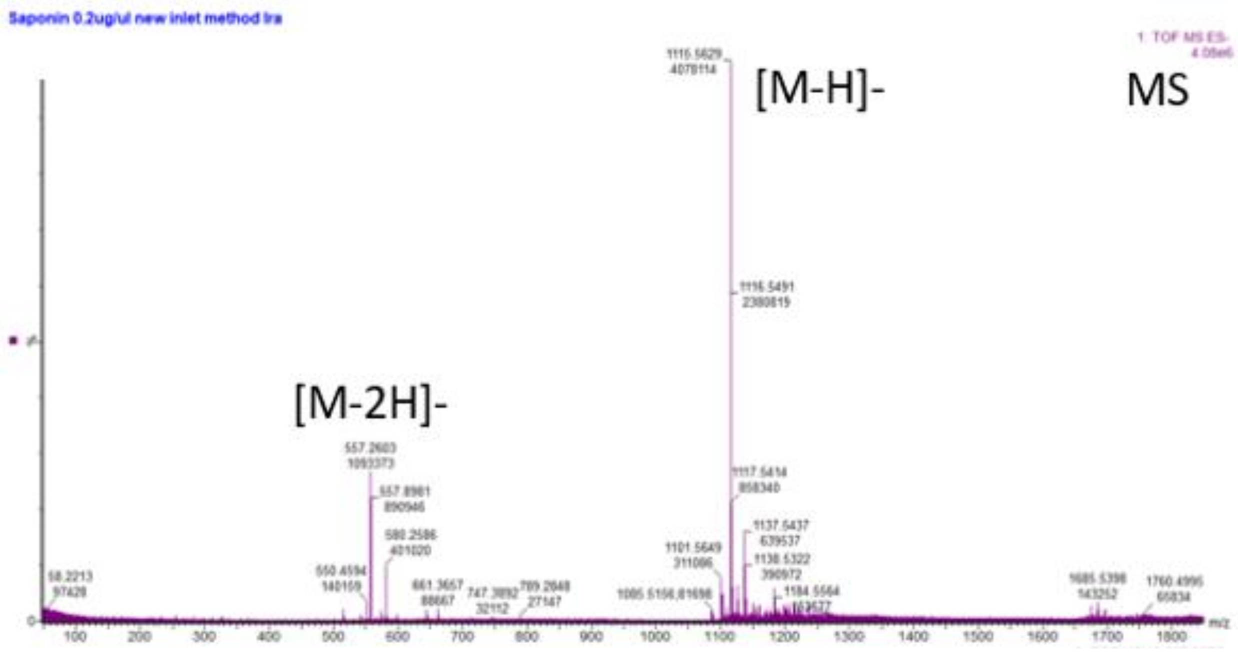

Figure S2: UHPLC-QToF-MS spectrum of silviridoside in the negative ion mode

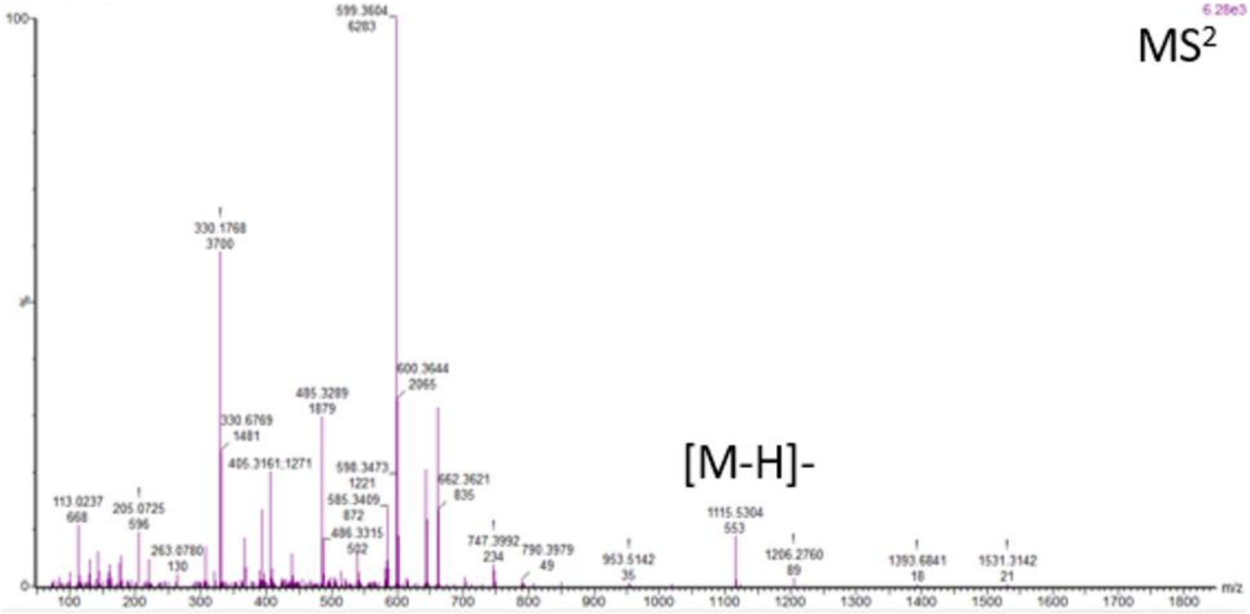

Figure S3: UHPLC-QToF-MS/MS spectrum of silviridoside in the negative ion mode

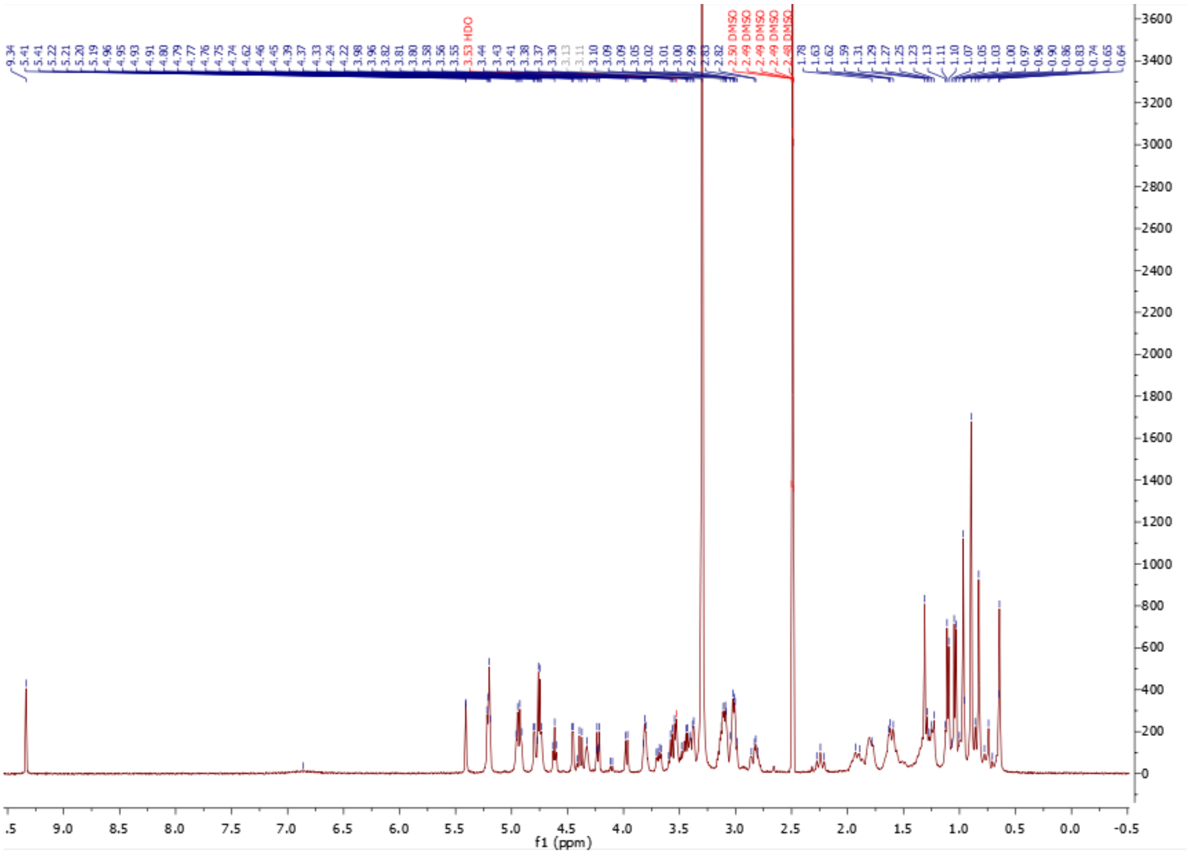

Figure S4: ^{1}H NMR spectrum of silviridoside

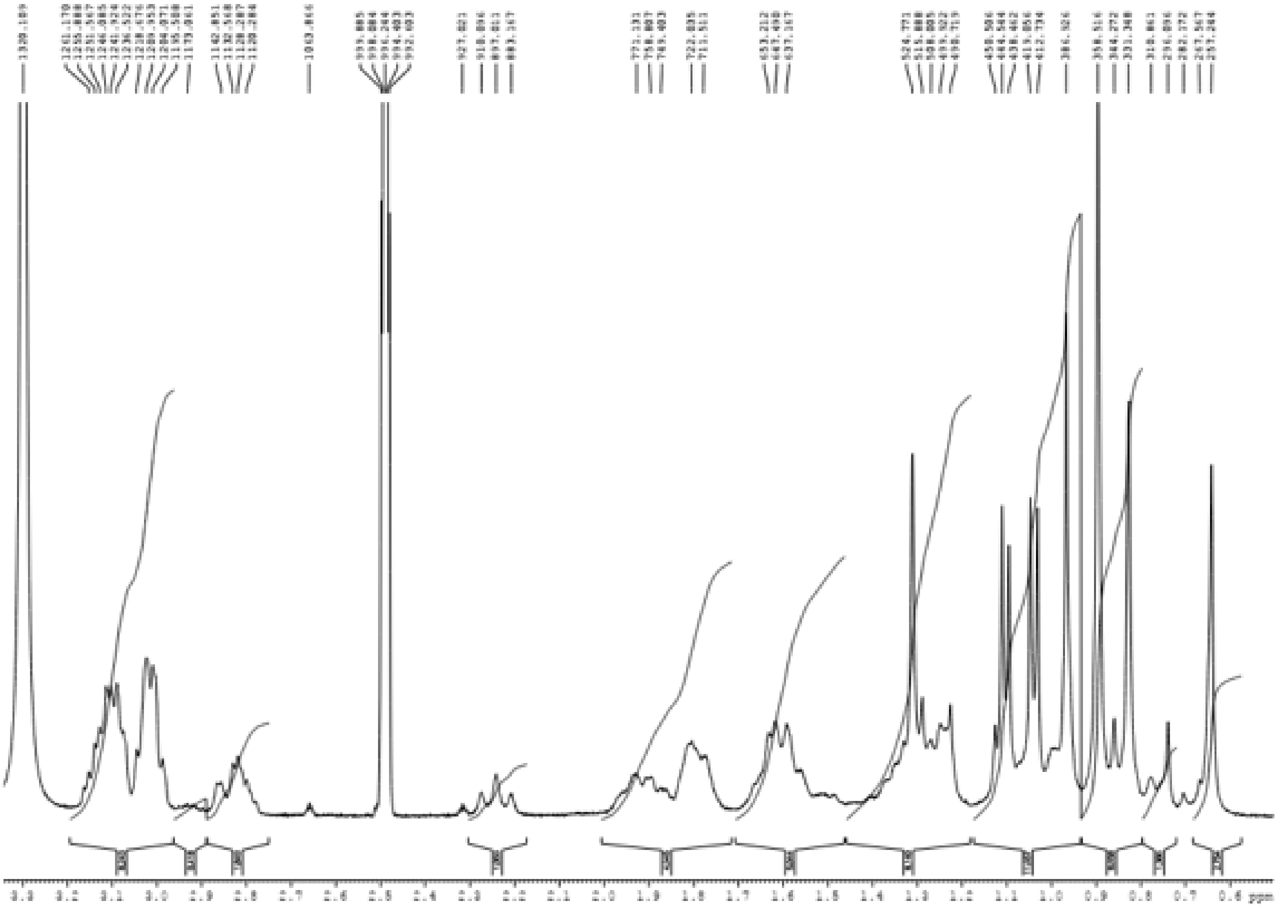

Figure S5: ^{1}H NMR spectrum of silviridoside (Expanded part)

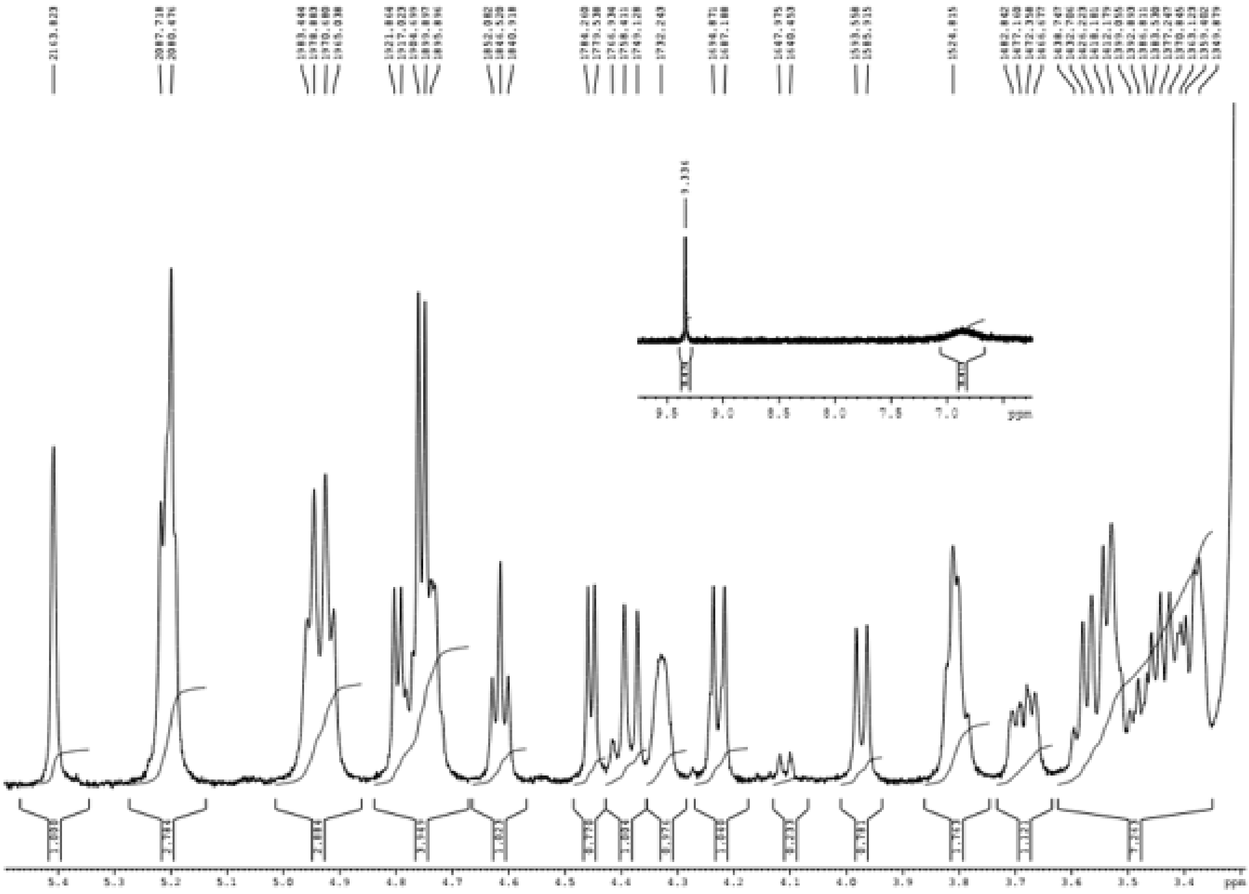

Figure S6: ^{1}H NMR spectrum of silviridoside (Expanded part)

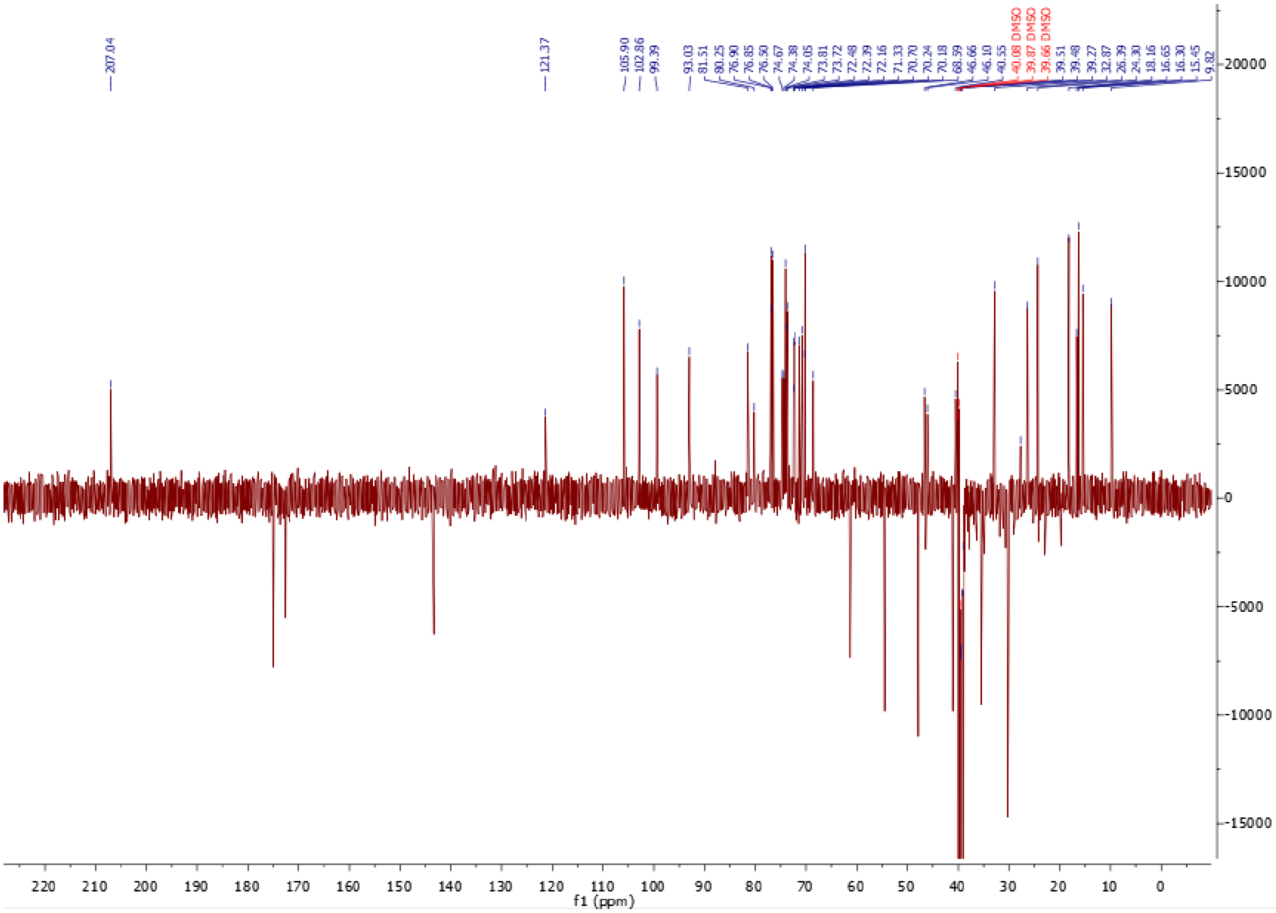

Figure S7: ^{13}C NMR spectrum of silviridoside

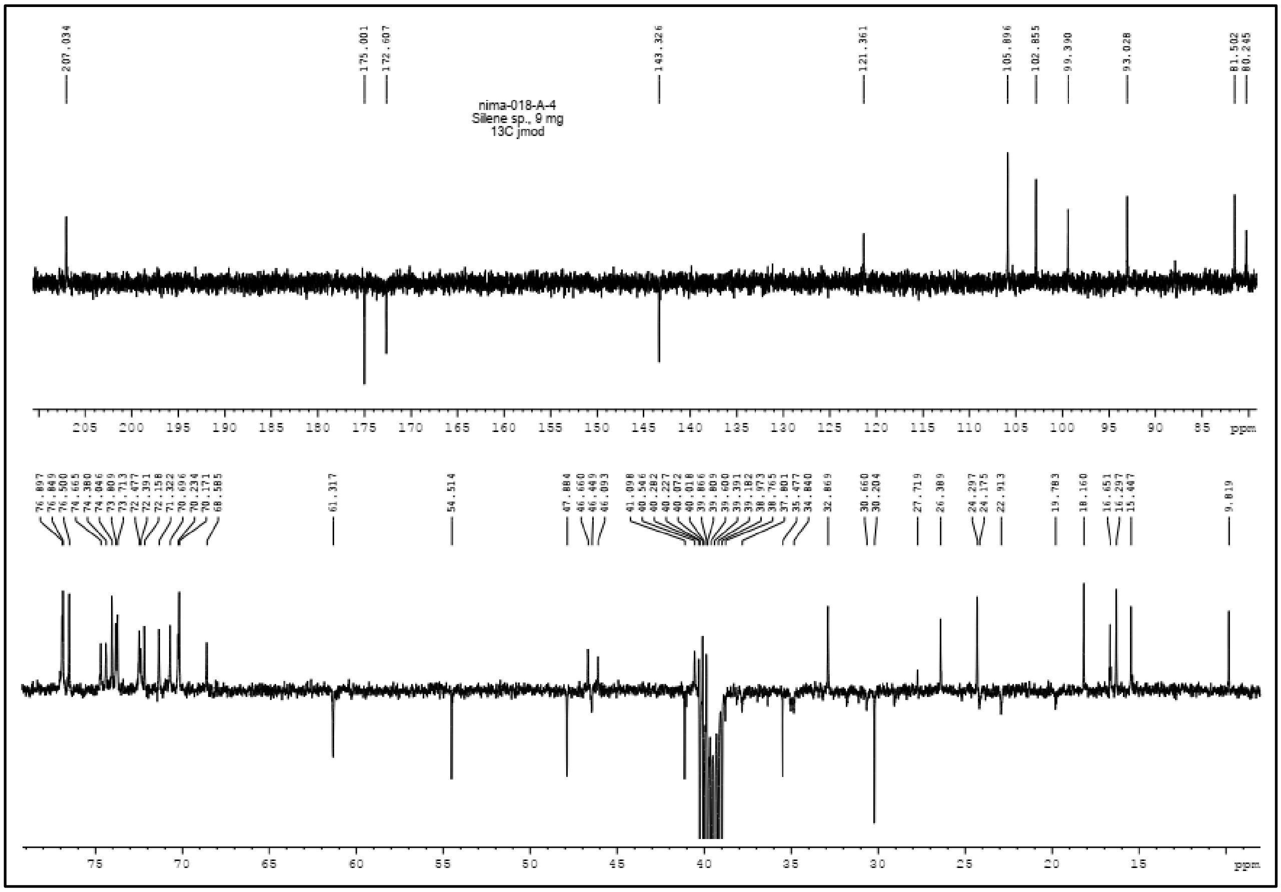

Figure S8: ^{13}C NMR spectrum of silviridoside - (Expanded part)

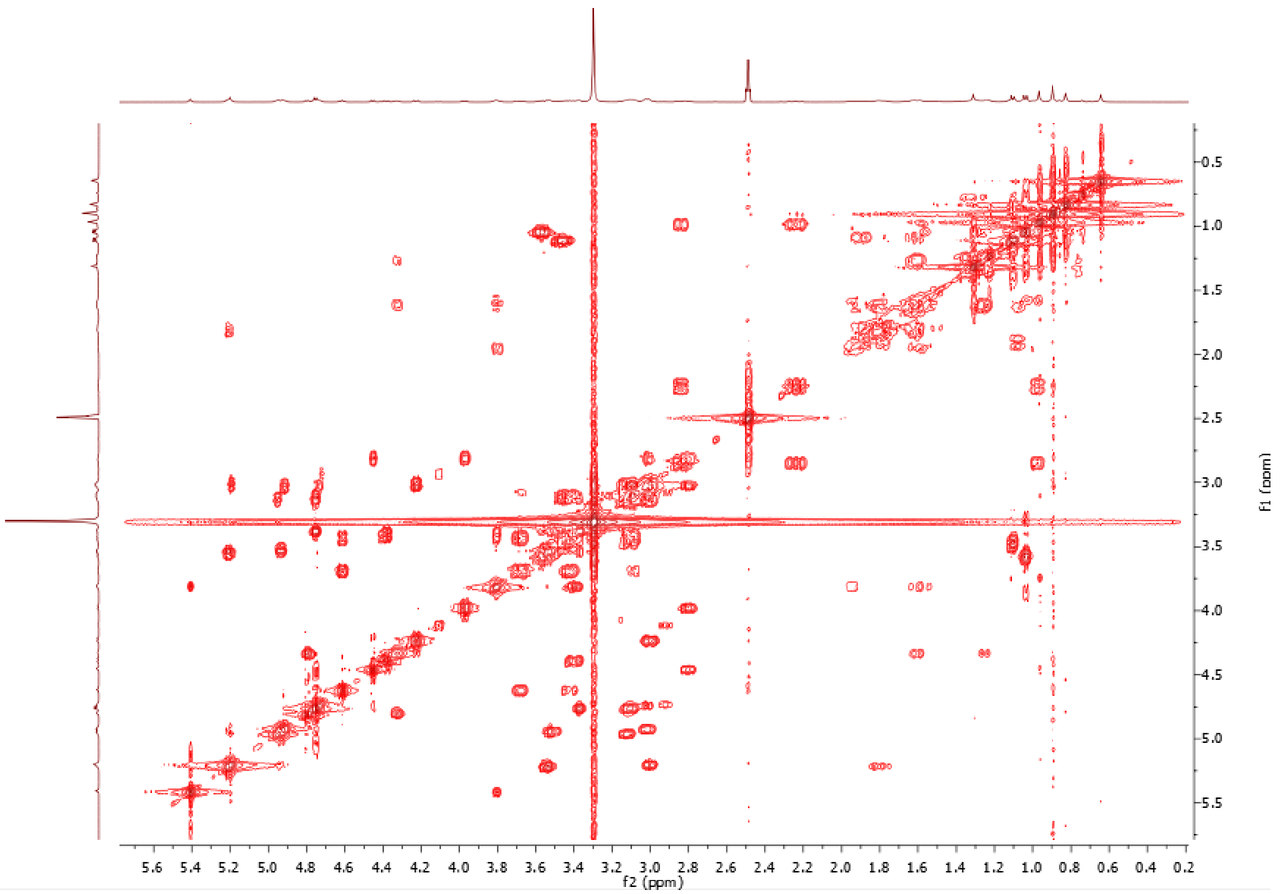

Figure S9: COSY spectrum of silviridoside

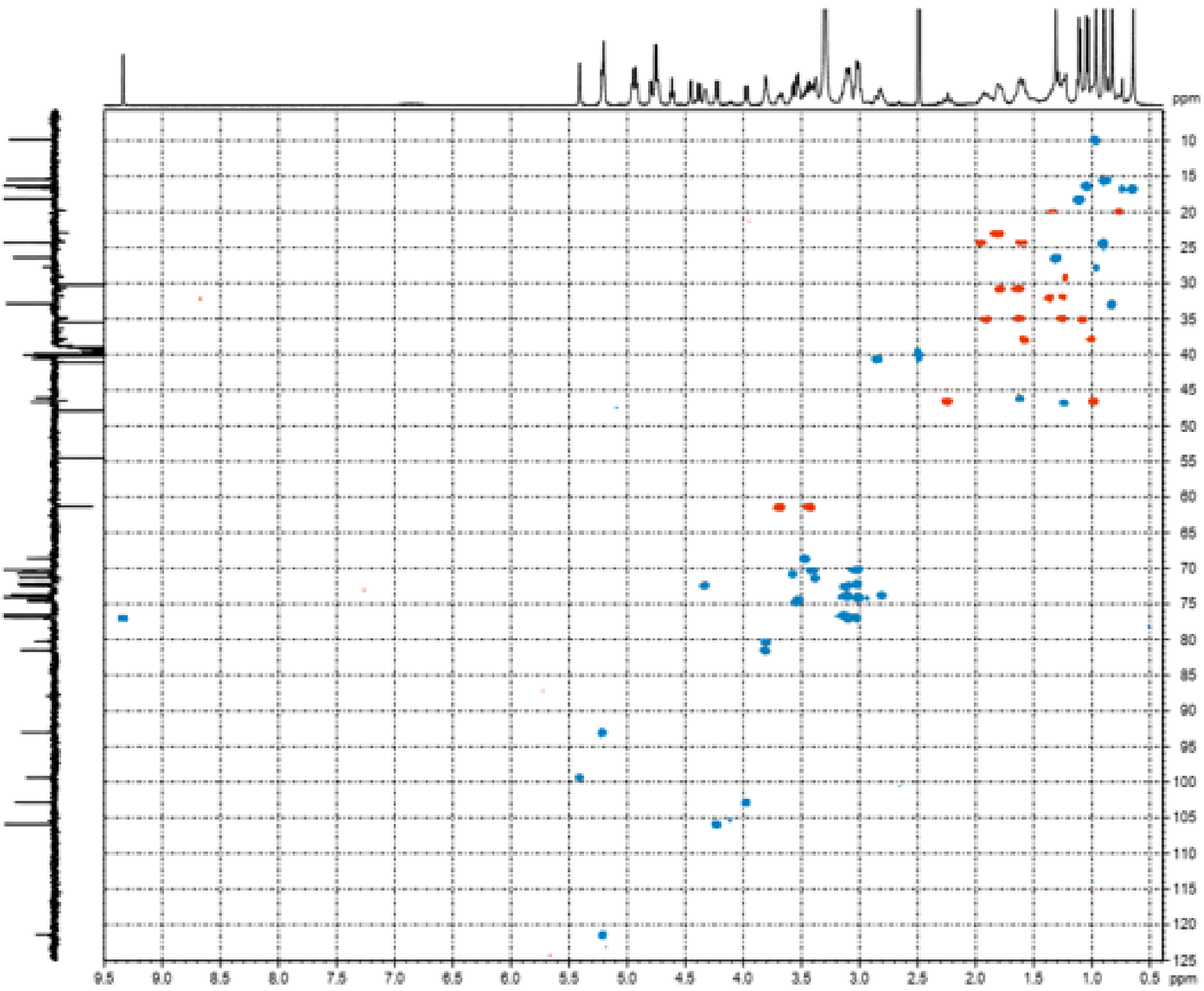

Figure S10: HSQC spectrum of silviridoside

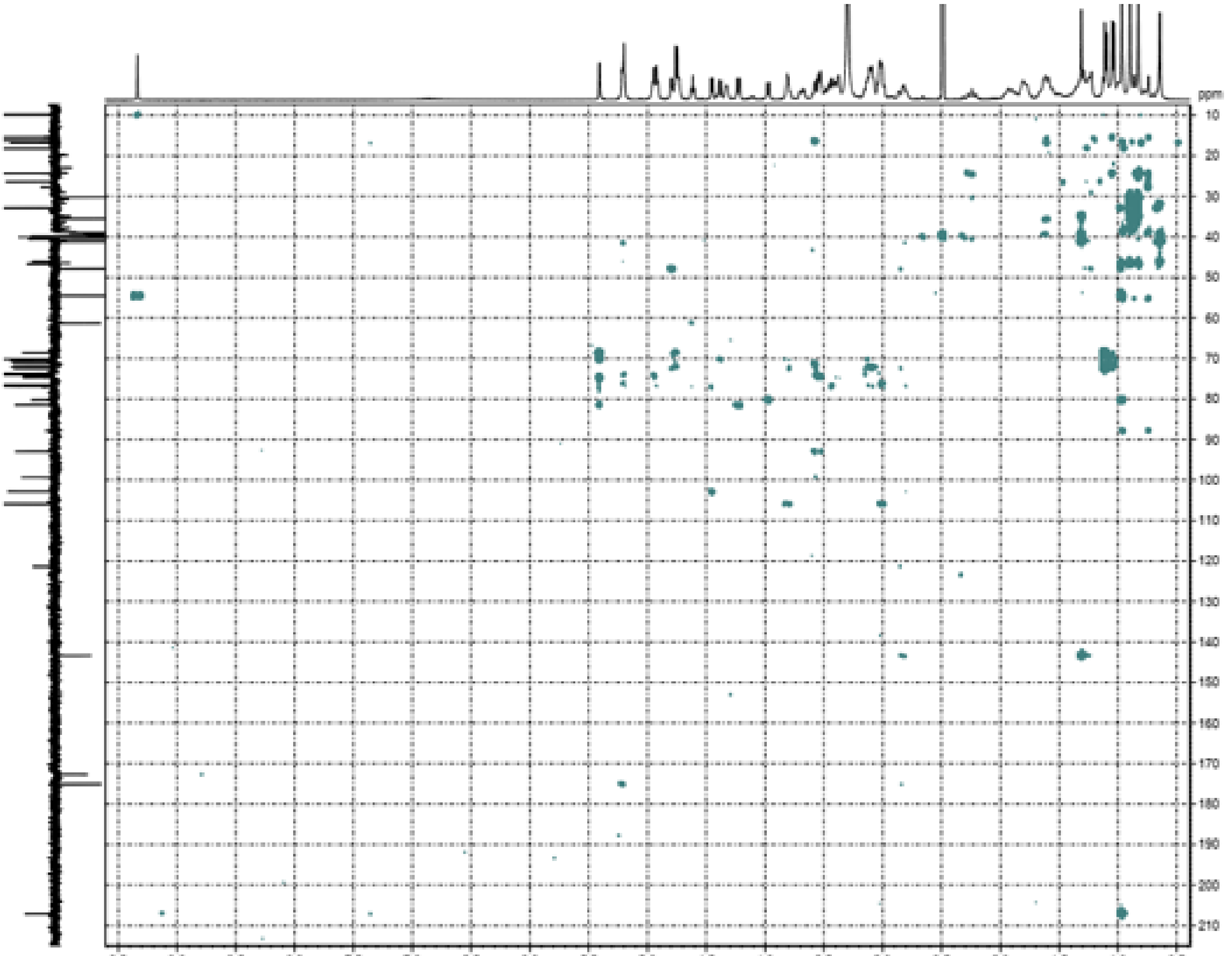

Figure S11: HMBC spectrum of silviridoside

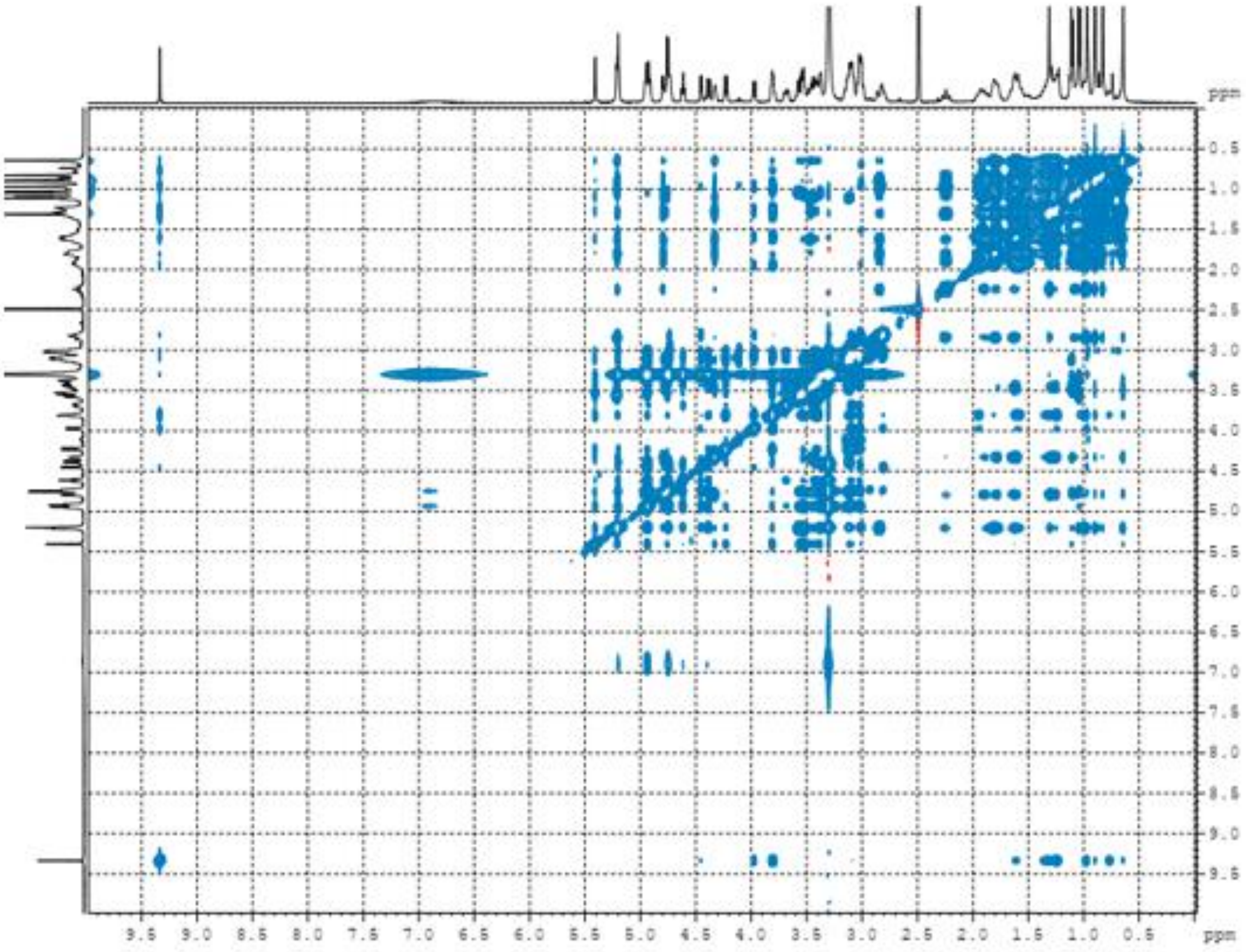

Figure S12: NOESY spectrum of silviridoside
